# Preclinical Animal Models in Facial Transplantation

**DOI:** 10.1097/GOX.0000000000002455

**Published:** 2019-09-23

**Authors:** Elie P. Ramly, Rami S. Kantar, Allyson R. Alfonso, J. Rodrigo Diaz-Siso, Eduardo D. Rodriguez

**Affiliations:** From the Hansjörg Wyss Department of Plastic Surgery, NYU Langone Health, New York, N.Y.

## Abstract

The technical feasibility and clinical applicability of facial transplantation (FT) have been demonstrated, yet animal models with different technical nuances and allograft compositions continue to be developed. We sought to provide a comprehensive appraisal of the current scope and value of animal models in FT.

**Methods:**

A comprehensive review of the literature was performed to identify all studies relevant to preclinical animal FT. Abstracts, texts, and references were screened. Both large and small animal models in studies including survival experimental arms were included. Purely anatomical or cadaveric animal studies were excluded, as were non-English language articles.

**Results:**

Twenty-nine unique models were identified, including 10 large (nonhuman primate, swine, and canine) and 19 small (rabbit, rat, and mouse) animal models. Orthotopic models were described in 70% of large and 73.7% of small animal studies. One study described a 2-stage rat FT model. Nerve coaptations were performed in 20.7% of all models (1 canine, 1 rabbit, and 4 rat models). One rat model allowed the study of both functional recovery and cortical reintegration of the allograft. Survival rates and immunological outcomes varied per model and protocol.

**Conclusions:**

A comprehensive review of animal models in FT shows redundancy spanning a variety of species, allograft compositions, and experimental designs. Although initial studies have focused on safety and technical feasibility, recent advances present specific opportunities for refining our understanding of functional and immunological challenges. As clinical experience continues to evolve, animal models may play an increasingly modest yet targeted role in FT.

## INTRODUCTION

Since the first human case in 2005, facial transplantation (FT) has become a viable reconstructive option for patients with extensive facial disfigurement not amenable to conventional approaches and can achieve excellent functional and aesthetic outcomes.^1^ Systematic team training, cadaveric simulation, and the use of advanced computerized surgical planning have culminated in the convergence of aesthetic, craniofacial, and microsurgical principles for reliable clinical outcomes.^2–4^ Rather than following a linear translational path from “bench to bedside,” clinical FT was made possible by the integration of experiences in upper extremity and solid organ transplantation, and decades of preclinical vascularized composite allotransplantation (VCA) research that had yet to deliver a replicable large animal FT model by the time the first human face transplant took place.

Preclinical animal models have provided an essential platform for technical, functional, and immunological advances in VCA.^5–8^ However, their current role in FT is worth revisiting as the field enters its second clinical decade. In this article, we provide a comprehensive review of preclinical animal models in FT, highlighting the features that remain imperative for the field’s progression, and others beyond which the field has already progressed.

## METHODS

A comprehensive review of the literature was performed to identify all articles relevant to preclinical animal FT, using the following search terms: “preclinical model,” “animal model,” “face transplant,” “facial transplantation,” and “vascularized composite allotransplantation.” Abstracts, texts, and references were screened. Original contributions and reviews describing VCA were screened for specific references to FT. Large and small animal species identified were again included in the search. Only studies describing a unique vascularized composite FT animal model including a survival experimental arm were included. Publications describing the replication of a previously described model without substantial modifications in allograft tissue composition or technical approach were excluded. Models only including a facial subunit such as the ear were excluded. Purely anatomical or cadaveric animal studies were excluded, as were non-English language articles.

## ANIMAL MODELS IN FT

Twenty-nine models were identified. There were 10 large animal models (Table 1), including 4 nonhuman primate (NHP), 2 swine, 4 canine models, and 19 small animal models (Table 2), including 5 rabbit, 13 rat, and 1 mouse models. For studies with >1 experimental arm, the tables only mention the allogeneic transplantation arms. Orthotopic models constituted 70% of large animal models and 73.7% of small animal models. One rat study described 2-stage FT models.^9^ Nerve coaptations were performed in 20.7% of all models, including 1 canine model, 1 rabbit, and 4 rat models. One rat model allowed the study of both functional recovery and cortical reintegration of the allograft.^10^

**Table 1.** Large Animal Survival Models in Vascularized Composite Facial Transplantation

Animal Model	Author, Year Location	Allograft Design	Anastomoses/Coaptations	Immunosuppression	Allograft Survival (d)
Nonhuman primate	Gold et al (1991)^14^ Orange, Calif.	Oromandibular (O) Bone, muscle, skin, mucosa	Vessels CCA, EJV (D; R) Nerves: no	Cyclosporine Methylprednisolone	13–65
	Silverman et al (2008)^15^ Baltimore, Md.	Oromandibular (H) Bone, muscle, skin	VesselsCCA, IJV (D) SFA, SFV (R) Nerves: no	Thymoglobulin Rapamycin Tacrolimus Methylprednisolone	6–129
	Barth et al (2009) Baltimore, Md.	Oromandibular (H) Bone, muscle, skin	Vessels CCA, EJV, and IJV (D) CFA, CFV (R) Nerves: no	Tacrolimus	60–177
	Barth et al (2011)^18^ Baltimore, Md.	Oromandibular (H) Group 1: bone, muscle, skin Group 2: muscle, skin	Vessels CCA, EJV, and IJV (D) CFA, CFV (R) Nerves: no	All animals: tacrolimus, mycophenolate mofetil	Group 1: 205–430 Group 2: 9–42
Swine	Kuo et al (2009)^20^ Taoyuan, Taiwan; Pittsburgh, Pa.	Hemiface (O) Cartilage (ear), muscle, parotid, nerve, skin	Vessels CCA, EJV (D; R) Nerves: no	Cyclosporine	38–49
	Park et al (2016)^22^ Seoul, Korea	Hemiface (O) Bone (maxillary, mandibular), cartilage (ear), muscle, parotid, nerve, skin	Vessels CCA, EJV (D; R) Nerves: no	None	7–10
Canine	Höhnke et al (1997)^23^ Pittsburgh, Pa.	Partial mandibular (O) Bone, “soft tissue”	Vessels Unreported (D) Un-named artery, JV (R) Nerves: no	Tacrolimus	14–912
	Eduardo Bermú Dez et al (2002)^24^ Bogota, Columbia	Hemiface (O) Muscle, skin	Vessels FA, EJV (D) LA, EJV (R) Nerves: no	Cyclosporine Prednisone	7
	Shengwu et al (2007)^25^ Shanghai, China	Upper hemiface (O) Group 1: cartilage (ear), muscle, nerve, skin Group 2: cartilage (ear), tarsal plate, conjunctiva, muscle, nerve, skin	Vessels ECA, EJV (D; R) Nerves: facial (D; R)	All animals: cyclosporine, Methylprednisolone or prednisone	29–402
	Lee and Eun (2014)^26^ Seoul, Korea	Hemiface (O)Cartilage (ear), muscle, parotid, nerve, skin	Vessels ECA, EJV (D; R) Nerves: no	Tacrolimus	7–10

CCA, common carotid artery; CFA, common femoral artery; CFV, common femoral vein; D, donor; ECA, external carotid artery; EJV, external jugular vein; FA, facial artery; H, heterotopic; IJV, internal jugular vein; JV, jugular vein; LA, lingual artery; O, orthotopic; R, recipient; SFA, superficial femoral artery; SFV, superficial femoral vein.

**Table 2.** Small Animal Survival Models in Vascularized Composite Facial Transplantation

Animal Model	Author, Year Location	Allograft Design	Anastomoses/Coaptations	Immunosuppression	Allograft Survival (d)
Rabbit	He et al (1990)^27^ Wuhan, China	Mandibular (O) Bone, skin	Vessels FA, FV (D; R) Nerves: no	Group 1: none Group 2: azathioprine, prednisone Group 3: cyclosporine	10 10 17.9–100
	Randzio et al (1991)^28^ Orange, Calif.	Mandibular (O) Group 1: bone (hemimandible), muscle, nerve, skin (cheek, lip) Group 2: bone (partial hemimandible), muscle, nerve, skin (cheek, lip)	Vessels CCA, EJV (D; R) Nerves Facial, inferior division (D; R) Mandibular (trigeminal) (D; R)	Cyclosporine	Unreported–100
	Xudong et al (2006)^29^ Xi’an, China	Hemiface (O) Cartilage (ear), muscle, skin	Vessels CCA, EJV (D) ECA, EMV (R) Arterial anastomosis Groups 1 and 2: end-to-end Group 3: end-to-side Nerves: No	Group 1: None Group 2: cyclosporine, prednisone, azathioprine Group 3: cyclosporine, prednisone, azathioprine	4–7 1–100 1–100
	Nie et al (2008)^30^ Harbin, China	Hemiface (O) Bone (calvaria), cartilage (ear), parotid, muscle, skin	Vessels CCA, EJV (D) CCA, PFV (R) Nerves: no	Group 1: none Group 2: cyclosporine, prednisone	0–10 18–120
	Baek et al (2010) Seoul, Korea	Hemiface (O) Cartilage (ear), muscle, skin	Vessels CCA, EJV (D: R) Nerves: no	None	2–9
Rat	Ulusal et al (2003)^31^ Cleveland, Ohio	Full face/scalp (O) Cartilage (ear), muscle, skin	Vessels CCA, EJV (D) ECA, FA, AFV (R) Nerves: no	Cyclosporine	0–172
	Demir et al (2004)^33^ Cleveland, Ohio	Hemiface (O) Cartilage (ear), parotid, skin	Vessels CCA, EJV (D; R) Nerves: no	Group 1: none Group 2: cyclosporine	5–7 171–240
	Yazici et al (2006)^34^ Cleveland, Ohio	Hemiface (O) Bone (calvaria), cartilage (ear), muscle, skin	Vessels CCA, EJV (D; R) Nerves: no	Cyclosporine	90–220
	Yazici et al (2007)^36^ Cleveland, Ohio	Maxillary (H) Bone, cartilage, teeth, muscle, mucosa, nerve	Vessels CCA, EJV (D) FA, FV (R) Nerves: no	Cyclosporine	1.5–105
	Landin et al (2008)^38^ Valencia, Spain	Hemiface (O) (with mystacial pad) Cartilage (ear), muscle, nerve, skin	Vessels CCA, EJV (D; R) Nerves Group 1: no Group 2: Zygomaticorbital, bucolabial, upper marginal mandibular (facial) (D; R) Infraorbital (trigeminal) (D; R)	Tacrolimus	0–56
	Washington et al (2009)^10^ Pittsburgh, Pa.	Hemiface (O) (with mystacial pad) Cartilage (ear), muscle, nerve, skin	Vessels CCA, EJV (D; R) Nerves Buccal, marginal mandibular (facial) (D; R) Infraorbital (trigeminal) (D; R)	Cyclosporine	140
	Kulahci (2010) Istanbul, Turkey; Cleveland, Ohio	Hemiface (H) Bone (mandible), cartilage (ear), teeth, muscle (tongue), mucosa, skin	Vessels CCA, EJV (D) FA, FV (R) Nerves: no	None (syngeneic)	350
	Zor et al (2010)^42^ Cleveland, Ohio	Midface (H) Bone (premaxilla), cartilage (nose), teeth, muscle, mucosa, nerve, skin	Vessels CCA, EJV (D) FA, FV (R) Nerves Facial (D) to femoral nerve (R) Infraorbital (D) to saphenous nerve (R)	Cyclosporine	100
	Lim and Eun (2014)^35^ Seoul, Korea	Hemiface (O) Cartilage (ear), skin	Vessels CCA, EJV (D; R) Nerves: no	Cyclosporine	2–14
	Ramirez et al (2015)^9^ Taoyuan, Taiwan	Hemiface Cartilage (ear), muscle, parotid and submandibular glands, lymph nodes, skin Group 1: single-stage (O) Group 2: local 2-stage (H/O) Group 3: distant 2-stage (H/O)	Vessels Groups 1 and 2 CCA, EJV (D) FA, EJV (R) Group 3 CCA, EJV (D) FeA, FeV then FA, EJV (R) Nerves: no	Group 1a: none Group 1b: cyclosporine Group 2a: none Group 2b: cyclosporine Group 3a: none Group 3b: cyclosporine	Mean: 14.3 ± 4.5 42 Mean: 18.5 ± 1 42 Mean: 14.3 ± 5.7 42
	Kulahci (2016) Ankara, Turkey; Chicago, Ill.	Hemiface (H) (with mystacial pad) Bone (premaxilla), cartilage (ear, nose), teeth, muscle, mucosa, skin	Vessels CCA, EJV (D) FA, FV (R) Nerves: no	Cyclosporine	100
	Gao et al (2017) Shanghai, China	Periorbital (H) Muscle, tarsus, skin	Vessels ECA, PFV (D) FA, FV (R) Nerves: no	None (syngeneic)	0–60
	Gao et al (2017) Shanghai, China	Periorbital (O) Muscle, tarsus, skin, nerve	Vessels ECA, PFV (D) CCA, EJV (R) Nerves Temporal and upper zygomatic (facial) (D; R)	None (syngeneic)	0–60
Mouse	Sucher et al (2012)^46^ Innsbruck, Austria; Baltimore, Md.; Pittsburgh, Pa.; Taipei, Taiwan	Hemiface (O) Cartilage (ear), muscle, skin	Vessels CCA, EJV (D; R) Nerves: no	None	Unreported

AFV, anterior facial vein; CCA, common carotid artery; D, donor; ECA, external carotid artery; EJV, external jugular vein; EMV, external maxillary vein; FA, facial artery; FeA, femoral artery; FeV, femoral vein; FV, facial vein; H, heterotopic; IMV, internal maxillary vein; O, orthotopic; PFV, posterior facial vein; R, recipient.

Significant heterogeneity in the publications precluded a quantitative analysis. Vessel size and size of transplanted tissue were not consistently reported. Across different studies, graft rejection was most commonly assessed using clinical observation and physical exam, with emphasis on signs of inflammation, ulceration, graft shrinkage, necrosis, and tooth or hair loss. The animals’ general health and weight fluctuations were not consistently reported. For large animals models, imaging and laboratory tests used included various combinations of radiography, Technetium 99 bone scans, magnetic resonance imaging, indocyanine green fluorescence angiography, electromyography, histology, immunologic assays, and peripheral blood tests such as hematocrit, white blood cell count, serum chemistry, tacrolimus levels, flow cytometry, and mixed lymphocyte reaction assay. For small animal models, assessment included radiography, computerized tomography scanning, Technetium 99 bone scans, angiography, histology, and/or immunohistochemistry, as well as flow cytometry to evaluate for chimerism and mixed lymphocyte reaction assay to determine donor-specific tolerance. Electroneurography, electromyography, and invasive cortical testing were occasionally used to evaluate functional outcomes. The cost of different animal models was not reported, but may vary by country and vendor, and depends on animal species, breed, and weight, with additional consideration given to housing and husbandry, veterinary services, and animal health testing. Procurement and housing costs of large animal models are generally more prohibitive than those of small models.^11^

### Large Animal Models

Large animal models for FT have been developed in NHP, swine, and canines (Table 1). Although they require more resources and present more challenges in terms of logistical management and husbandry, large animal models offer obvious advantages in virtue of their anatomical, physiological, and immunological similarity to humans.^12,13^

#### Nonhuman Primates

Gold et al^14^ described the first orthotropic vascularized hemimandibular (bone, muscle, skin, and mucosa) allotransplant model in outbred cynomolgus monkeys. The 4 recipients had limited survival under cyclosporine immunosuppression, and pathological specimens showed fibrosis and degeneration of the allografted dermis, skeletal muscle, and marrow cavities. Despite rejection in all grafts, the 2 longest survivors were reported to have the ability to chew, tolerate a diet, and gain weight.

In 2008, the senior author (E.D.R.) and collaborators described a heterotopic NHP model.^15^ The composite allograft did not include mucosa to avoid additional antigenicity and secretions on the surgical site. The pedicle was based on the common carotid artery and internal jugular vein, and the allograft was transplanted to the recipient’s groin at the superficial femoral artery and vein. Treatment consisted of thymoglobulin, rapamycin, and tacrolimus. There were frequent immunosuppression-related complications, early graft losses, rejection, and variable survival rates. Allograft histology in the longest-term survivor showed dermal lymphocytic infiltration and extensive fibrosis, viable muscle with mild myositis, and healthy cartilage and bone elements.

**Fig. 1.**
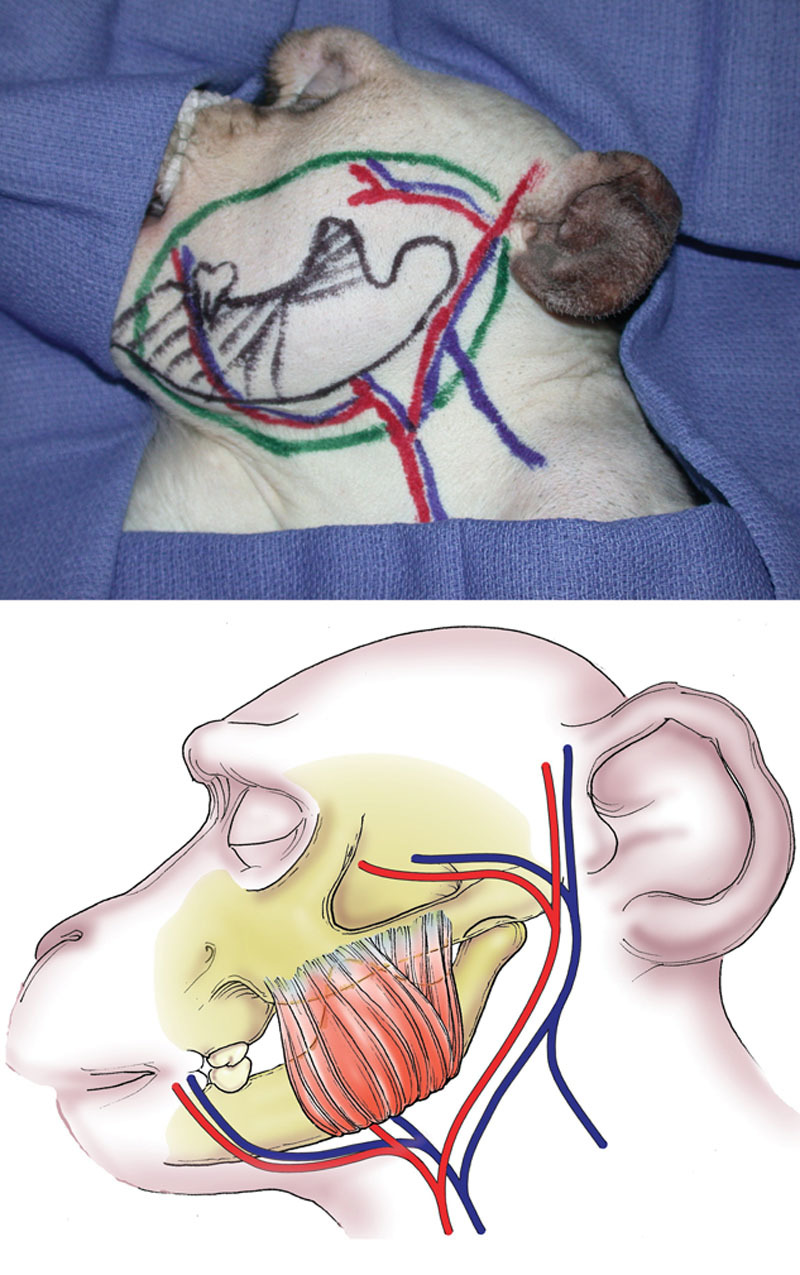
Heterotopic partial face transplantation in a nonhuman primate model. Donor composite facial graft photographs (above) with markings and schematic drawing (below). The osteomyocutaneous facial segment is based on the common carotid artery and both jugular veins and includes the facial, transverse facial, and superficial temporal arteries. Reprinted with permission from *Plast Reconstr Surg 2009;123:493–501*.

**Fig. 2.**
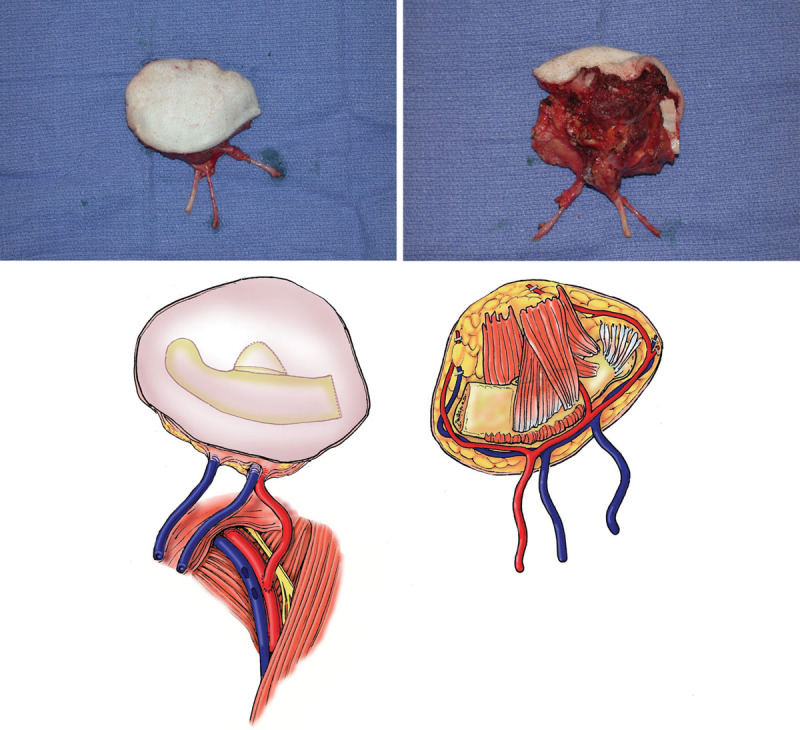
Heterotopic partial face transplantation in a nonhuman primate model. Intraoperative photographs (above) and schematic drawings (below) of facial subunit depicting bone, muscle, and skin; the common carotid artery; and the internal and external jugular veins. Reprinted with permission from *Plast Reconstr Surg 2009;123:493–501.*

**Fig. 3.**
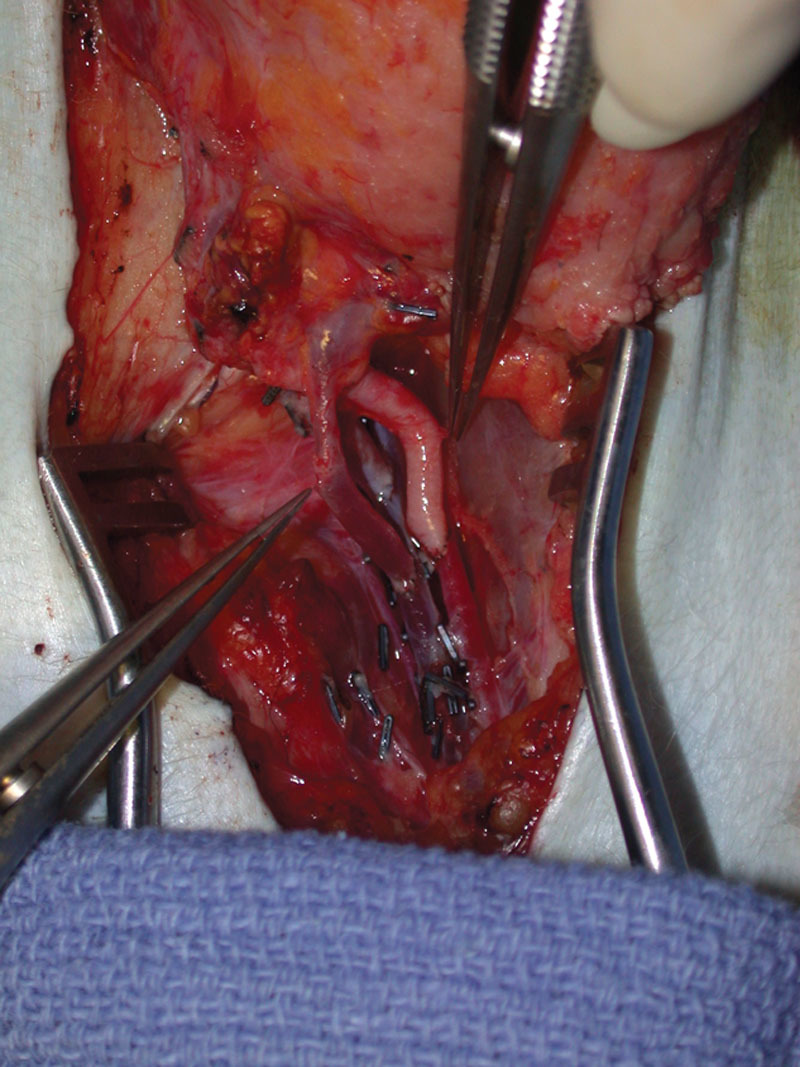
Heterotopic partial face transplantation in a nonhuman primate model. The allograft is heterotopically transplanted to the recipient, with vascular anastomoses performed to the femoral vessels (arterial anastomosis: common carotid artery to common femoral artery, end-to-side); dual venous anastomoses (internal and external jugular veins to common femoral vein, end-to-side). Reprinted with permission from *Plast Reconstr Surg 2009;123:493–501.*

**Fig. 4.**
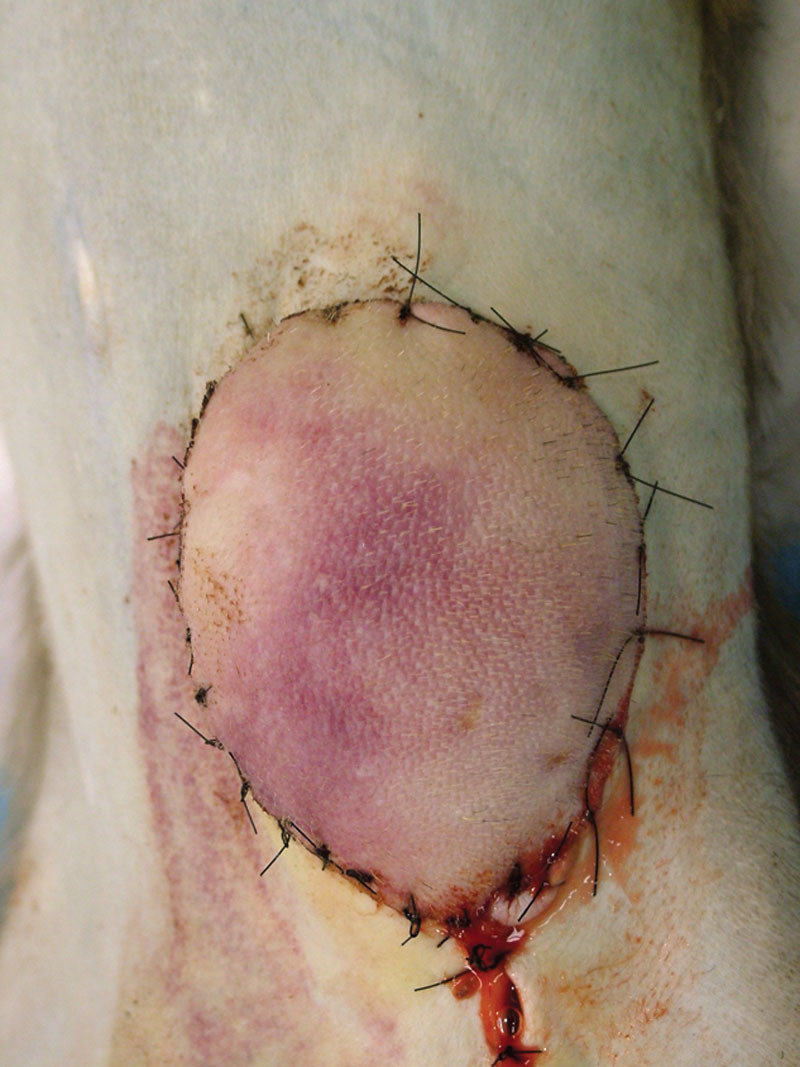
Heterotopic partial face transplantation in a nonhuman primate model. The graft is inset into the lower abdominal wall and sutured to the surrounding skin. This figure shows a graft that failed on postoperative day 3. Venous congestion is apparent. Reprinted with permission from *Plast Reconstr Surg* 2009;123:493–501.

In 2009, the team introduced modifications^16^ that led to the first reliable NHP FT model with prolonged rejection-free graft survival without early complications. The recipients received tunneled central venous catheter insertion the day before surgery for continuous infusion of tacrolimus for 28 postoperative days, subsequently tapered to daily intramuscular injections. End-to-side anastomosis was performed between the donor common carotid artery and recipient common femoral artery. The donor internal and external jugular veins were anastomosed in an end-to-side fashion to separate sites on the recipient’s common femoral vein (Figs. 1–4). The use of this dual venous outflow prevented early graft loss from venous congestion and thrombosis. Tacrolimus monotherapy was well tolerated with no major side effects or metabolic derangements. Some of the recipients developed line-related infections. Five animals had no serious complications, no graft failure, and no clinical evidence of graft rejection but developed post-transplant lymphoproliferative disease (PTLD). Subsequent genotypic analysis showed that 3 (60%) of those animals demonstrated tumors of majority donor genotype and 2 animals revealed recipient-derived tumors.^17^ The high frequency of donor-derived PTLD raised suspicion for whether the transplantation of a large volume of vascularized bone marrow (VBM) in composite tissue allografts may be a risk factor for PTLD.

Experiments were performed with and without inclusion of the donor mandibular segment to investigate the immunomodulatory role of VBM.^18^ Four recipients receiving oromandibular composite allografts including VBM were compared with 3 receiving facial myocutaneous allografts missing the VBM component. The animals received no conditioning radiation or T-cell depletion. Tacrolimus and MMF were used for immunosuppression. The VBM group had prolonged graft survival (205–430 days) and demonstrated variable low-level macrochimerism and donor cell engraftment in recipient tissues, but rejection and graft loss did occur after discontinuation of immunosuppression. Myocutaneous allografts missing VBM all experienced early rejection and graft loss by postoperative days 7–15. To further investigate the role of bone marrow in potentially inducing tolerance, a small sample of animals receiving facial allografts without VBM were administered donor BMC infusion.^19^ The results, however, were discouraging. Grafts with BMC infusion underwent early rejection and no animal with infused BMC demonstrated any evidence of chimerism.

#### Swine

Kuo et al^20^ developed an orthotropic hemiface transplantation model in miniature swine with the common carotid artery and external jugular vein as the vascular pedicle. The model included the greater auricular sensory nerve but not the facial nerve or innervated muscle, and no nerve coaptation was done, preventing the assessment of functional outcomes. In a subsequent experiment,^21^ recipients receiving infused bone marrow–derived mesenchymal stem cells combined with cyclosporine treatment had significantly prolonged allograft survival compared with those who did not, with potentially associated modulation of T-cell regulation and cytokine expression.

Park et al^22^ described an orthotopic swine training model where 2 surgical teams operated simultaneously, 1 performing donor allograft procurement and the other preparing the recipient. The allograft included skin and subcutaneous fat, muscle, maxillary and mandibular bone (via Le Fort-I and mandibular osteotomies), parotid gland, and facial nerve, but the nerve was not repaired. Postoperative immunosuppression was not administered and acute rejection and flap necrosis occurred by days 7–10.

#### Canine

Höhnke et al^23^ reported vascularized composite partial mandibular allotransplantation between nonrelated Beagle dogs, with prolonged survival of up to 2.5 years on tacrolimus. The report lacks detailed information on donor and recipient vessels. The grafts were noted to be histologically viable (unknown time points) and no signs of infection or bone instability were noted in the long-term survivors. In 2002, Eduardo Bermú Dez et al^24^ described an orthotopic canine FT model consisting of a musculocutaneous flap dissected under the superficial musculoaponeurotic system and including the orbicularis oculi. The pedicle consisted of the facial artery and external jugular vein and recipient vessels were the lingual artery and external jugular vein; the recipient received cyclosporine and prednisone, but the flap was acutely rejected and the dog euthanized by postoperative day 7.

Shengwu et al^25^ described bilateral and unilateral facial composite flap designs. Two teams operated simultaneously on the donor and recipient dogs, and different flaps were performed as either auto- or allotransplants in 5 different experimental arms. For allotransplants, cyclosporine and steroids were used for immunosuppression. The 2 allotransplantation models, depicted in Table 1, included the unilateral superior half of the face and scalp, with modifications in tissue composition. The operative procedure included facial nerve coaptation, and the flaps included the nervus auriculopalpebralis and orbicularis oculi muscle. New action potentials were detected at 12 weeks on electromyography, and were more significant at 6 months, with gradual increase in amplitude. This correlated with eyelid closure reflex testing. Lee and Eun^26^ described another canine orthotopic model in 2014, with minor technical modifications but no nerve coaptation, and using tacrolimus as opposed to cyclosporine. Acute rejection and necrosis occurred within 7–10 days.

### Small Animal Models

FT has been more frequently attempted in small animals, including rabbits, rats, and mice (Table 2). Small animal care is less labor and resource-intensive and allows for a greater number of experiments, as well as a wider opportunity for immunological manipulation. However, their small size can make surgical reproducibility more challenging.^5^

#### Rabbit

He et al^27^ performed mandibular composite allotransplantation in rabbits, comparing the absence of immunosuppression to the administration of azathioprine with prednisone, or cyclosporine monotherapy at different doses. Allograft survival ranged from 10 days in the absence of immunosuppression or the use of azathioprine/prednisone to up to 100 days with higher doses of cyclosporine.

Randzio et al^28^ performed hemimandibular transplants in 41 rabbits (Table 2) and reported repairing the mandibular nerve and inferior division of the facial nerve, but sensory and motor regeneration was not thoroughly evaluated. The majority of allografts demonstrated wound healing and hair growth, and evidence of mandibular bone and dental growth was observed. Complications included venous and arterial thrombosis, abscess formation, necrosis, and rejection, as well as cyclosporine-related wasting syndrome.

Xudong et al^29^ described an orthotopic hemifacial model and compared end-to-end versus end-to-side arterial anastomoses: thrombosis occurred in both groups and there was no apparent difference in allograft survival rates. Episodes of acute rejection were observed, as was fatal immunosuppression-related anorexia. Nie et al^30^ introduced calvaria to the model. The experimental design included anatomical studies, followed by implementation in an autograft group and allograft treatment and control groups. In the treatment group receiving cyclosporine and prednisone, rejection-free survival of up to 120 days was achieved with viable bone on biopsy.

#### Rat

Rats are the most frequently used animals in the study of FT (Table 2). The first model was described by Ulusal et al^31^ and Siemionow et al^32^ and consisted of the orthotopic transplantation of a full face/scalp and ears composite allograft. Subsequent studies introduced hemifacial iterations.^33–35^ Cyclosporine monotherapy permitted long-term allograft survival and donor-specific chimerism was observed, maintained by CD4 and CD8 T-cell subpopulations. Other modifications in allograft design and tissue composition were described^36^ (Table 2), extending from musculocutaneous and cartilage-containing flaps to more complex designs, including parotid gland, bone, teeth, and mucosa. While establishing technical feasibility and allowing immunological investigation, the early rat models did not address functional outcomes.

Landin and Cavadas^37^ described the “mystacial pad flap.” The mystacial pad is the specialized facial functional unit in the rat that contains the vibrissae (whiskers) which perform the rhythmic sweeping or “whisking” used by the animal to explore its environment. This has become a chosen feature for the study of motor and sensory function in rat FT, as it can be transplanted and monitored without jeopardizing vital facial functions.^5^ Landin et al^38^ developed an orthotopic hemifacial-mystacial pad transplant model to study functional recovery. Coaptations were performed for the zygomaticorbital, bucolabial, and upper marginal mandibular branches of the facial nerve, as well as the infraorbital branch of the trigeminal nerve. In contrast to other rat cyclosporine-based protocols, the authors used tacrolimus immunosuppression, for its desirable effects on nerve regeneration.^39,40^ They reported evidence of clinical, neurophysiological, and histological neural recovery.^38^ Washington et al^10^ extended the use of the model to study both functional outcomes and cortical reintegration. In the rat somatosensory cortex, each individual whisker can be correlated to a corresponding cortical area, providing an anatomical map that can allow for the study of cortical reafferentation through stimulation of the whiskers.^5,41^ All animals undergoing motor and sensory nerve coaptation showed physical and electrical evidence of motor function return and evidence of reafferentation on recording from the somatosensory cortex after whisker stimulation.^10^ A different heterotopic midface model by Zor et al^42^ included facial to femoral and infraorbital to saphenous nerve coaptations. At 100 days post-transplant, somatosensory-evoked potential and motor-evoked potential testing revealed that sensory and motor recovery reached 67% of normal latency values for infraorbital nerve and 70% for facial nerve latency values. Gao et al^43,44^ described heterotopic and orthotopic composite periorbital models, the latter including temporal and upper zygomatic branch coaptations, with subsequent signs of functional recovery.

Table 2 features other models that have diversified the technical applicability of FT in rats. Ramirez et al^9^ presented a particularly novel 2-stage approach to FT. Orthotopic transplantation was performed either in a single-stage approach, “distant 2-stage” (heterotopic transplantation to the groin as a first stage, followed by free flap transfer to the face on postoperative day 2), or “local 2-stage” (heterotopic transplantation to the neck as a first stage, followed by graft rotation as a pedicled flap to cover the facial defect on postoperative day 2). The allogeneic treatment arms of the study received cyclosporine and survived the full 42 days of follow-up. The staged approach is presented as an alternative to immediate transplantation in clinical situations where the condition of the wound or the general state of the patient preclude safe transplant or replantation, requiring procurement, revascularization, and banking of the composite flap at a heterotopic site until the opportune clinical conditions are established. The study’s initial results were encouraging with no significant difference in allograft survival or acute rejection grading between the 3 approaches. This may have potential future implications given a recent clinical report of the first immediate FT.^45^

#### Mouse

The development of reliable mouse models has traditionally been delayed by the technical challenges of microvascular anastomosis in particularly small, thin-walled vessels. Recent advances have enabled the development of FT in the mouse: In 2012, Sucher et al^46^ described the first mouse hemiface VCA model, consisting of skin, muscle, and ear cartilage, performed using a supermicrosurgical technique. The vascular pedicle consisted of the common carotid artery and the external jugular vein and the graft was transplanted orthotopically and revascularized by anastomoses to the corresponding recipient vessels using superfine microsurgical instruments and a nonsuture cuff technique. No nerve coaptation was performed. The authors reported a success rate of 78% after an initial learning curve. No immunosuppression was administered, and allograft rejection occurred in the allogeneic group within 14 ± 2 days.

## FUTURE DIRECTIONS

Face transplantation has gained wide recognition as a viable reconstructive option in patients with disfigurement not amenable to autologous reconstructive approaches. Yet, animal models continue to be developed. The value of highlighting the full array of animal models is two-fold. First, it provides a detailed inventory of established models to inform future efforts, minimizing redundant or wasteful experimentation. Second, it sheds light on specific aspects of FT that no longer warrant investigational reiteration in the preclinical setting. Questions of anatomy, feasibility, and surgical technique as applicable to humans are largely addressed by the field’s existing cadaveric resources and clinical experience. Other important features such as aesthetic outcomes and skull base considerations are not shared between humans and experimental animal models. Current clinically relevant challenges revolve around better addressing the risks of immunosuppression, achieving donor-specific tolerance, and optimizing nerve regeneration. Further investigations have to acknowledge both the advantages and limitations of preclinical experimentation and to incorporate recent advances in clinical FT to deliver translatable innovation. Functional models based on the mystacial flap pad may offer a promising avenue for improving our understanding of nerve regeneration and cortical reintegration in FT recipients. However, corresponding clinical advances in functional rehabilitation would need to be used as the starting point for such future experiments. In immunological experimentation, small rodents are typically desired for their short lifespans, accelerated reproductive cycles, and the availability of inbred and knockout strains as well as monoclonal antibodies and molecular probes.^5^ However, rodent experimentation does not readily translate into successful tolerance-induction strategies in outbred large animals or humans. Swine and NHP display major histocompatibility antigens with similarity to humans, giving them superiority to small animals in preclinical studies. However, they are exposed to less controllable environmental factors over a longer lifespan, and their complex immunology and higher immunosuppressive requirements make the reproducibility of such strategies challenging, and medication-induced toxicity more limiting.^47^ Recent developments in clinical FT immunology highlight innovative approaches that may stimulate future advances.^48^ As the field continues to develop, future breakthroughs in FT will rely on multidisciplinary efforts that combine the lessons learned from focused investigations with the breadth of knowledge gained from a maturing global clinical experience. Any further animal experimentation would have to focus on reproducibility and clinical applicability rather than technical novelty, to answer relevant questions that are in line with the current state of clinical FT and its challenges.

## CONCLUSIONS

A comprehensive review of all animal models in FT shows redundancy spanning a variety of species, allograft compositions, and experimental designs. Modern advances in clinical FT obviate the need for further development of animal models that focus primarily on surgical feasibility. However, the knowledge accumulated may be combined with clinical experience to refine our understanding of functional and immunological challenges. As clinical experience continues to evolve, animal models may play an increasingly modest yet targeted role in FT.
